# Sugar Functionalized Collagen Material for Local Modulation of Innate Immunity

**DOI:** 10.1002/advs.202415364

**Published:** 2025-05-24

**Authors:** Francesca Taraballi, Claudia Corbo, Julia Enterria‐Rosales, John Otto Martinez, Silvia Minardi, Laura Pandolfi, Xing Wang, Ennio Tasciotti, Kavindra V. Singh, Cesar A. Arias, Bruna Corradetti

**Affiliations:** ^1^ Center for Musculoskeletal Regeneration Houston Methodist Academic Institute Houston TX USA; ^2^ Orthopedics and Sports Medicine Houston Methodist Hospital Houston TX USA; ^3^ School of Medicine and Surgery Nanomedicine Center Nanomib University of Milano‐Bicocca Monza Italy; ^4^ IRCCS Istituto Ortopedico Galeazzi Milan Italy; ^5^ Center for Precision Environmental Health Baylor College of Medicine Houston TX USA; ^6^ Human Longevity Program IRCCS San Raffaele Roma Rome Italy; ^7^ Department of Human Sciences and Quality of Life Promotion San Raffaele Roma Open University Rome Italy; ^8^ Center for Infectious Disease Houston Methodist Research Institute Houston TX USA; ^9^ Division of Infectious Diseases and Department of Medicine Houston Methodist Hospital Houston TX USA; ^10^ Department of Medicine Weill Cornell Medical College New York NY USA; ^11^ Department of Medicine Section Oncology/Hematology Baylor College of Medicine Houston TX USA

**Keywords:** immunomodulatory patch, innate immunity, mannose, sugar functionalization

## Abstract

Small alterations during the early stages of the innate immune response to an implant can drive large changes in adaptive immunity. Biomaterials for regenerative purposes can be engineered to modulate this immune response in beneficial ways. This study presents an innovative patch designed and functionalized to target the innate immunity at the implant site. Mannose moieties are incorporated into collagen patches, resulting in a technology called Local Immunotuning Patch (LIP), designed to directly interact with antigen presenting cells through their mannose receptor. In vitro, LIP shows anti‐inflammatory effects on bone marrow‐derived macrophages and inhibitory properties even on methicillin‐resistant bacterial strains. Subcutaneous implantation in mice reveals that LIP modulates multiple pathways related to innate and adaptive immunity, underscoring its role in shaping an immune‐engineered environment around the implant. These findings highlight the potential of this strategy to control the foreign body reaction at the implant site, making it applicable for various uses, including wound healing and surgical infection control in reconstructive procedures.

## Graphical abstract

Modulation of the innate immune response at the implant site can significantly influence adaptive immunity. A mannose‐functionalized collagen patch, termed the Local Immunotuning Patch (LIP), has been designed to engage mannose receptors on antigen‐presenting cells while exhibiting antibacterial properties. LIP orchestrates complex immune pathways that promote tissue repair. This study underscores its potential to mitigate foreign body reactions and enhance tissue integration in regenerative medicine applications.

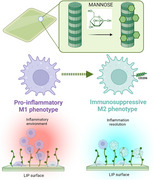

## 1 Introduction

Biomaterials serve as indispensable tools in numerous regenerative medicine applications.^[^ ^1^ ^]^ Among these, implantable bulk scaffolds hold significant promise, offering spatial organization for the growth of functional tissues and providing mechanical stability that supports cell adhesion and migration. Moreover, bulk scaffolds can be functionalized with bioactive molecules, such as growth factors or cytokines,^[^ ^2^ ^]^ to further enhance the regenerative process by promoting angiogenesis, reducing inflammation, and guiding tissue remodeling. The design of biomaterials has advanced from simple constructs that mimic target tissues’ structural and mechanical properties to sophisticated materials embedded with instructive signals aimed at multiple cellular targets. Despite many approaches developed to stimulate regeneration, many fail to investigate a crucial aspect: the direct targeting of key inflammatory players in the early stages of wound healing.^[^ ^3^ ^]^ This represents a significant gap in the field, as tuning the early inflammatory response may offer a more effective route to optimizing tissue repair and reducing fibrosis or scarring.^[^ ^4^, ^5^ ^]^ Carbohydrates, whether in the form of complex polysaccharides or conjugated to structural and functional proteins, are key components of the extracellular matrix (ECM), playing crucial roles in cell–environment interactions and cell–cell communication.^[^ ^6^ ^]^ Due to their chemical nature, carbohydrates are ideal candidates for biomaterial surface functionalization,^[^ ^7^, ^8^ ^]^ offering dual functionality^[^ ^9^ ^]^: they confer hydrophilic properties to the surface to which they are attached and are naturally recognized by the innate immune system. This natural interaction between sugars and immune cells suggests that functionalizing biomaterials with sugar‐based moieties could offer a novel and efficient means of directly influencing the immune system to enhance the regenerative response.^[^ ^4^ ^]^

Mannose, a monomeric carbohydrate, is critical in protein glycosylation and immune recognition. Notably, it has emerged as a promising alternative to antibiotics for treating urinary tract infections (UTIs) by inhibiting bacterial adherence to urothelial cells.^[^ ^10^ ^]^ The mannose receptor (CD206 or *Mrc‐1*), predominantly found on macrophages (MΦ) and dendritic cells (DC), recognizes carbohydrates on the cell walls of pathogens.^[^ ^11^ ^]^ Despite its significant role in biological processes, targeting the mannose receptor on MΦ has been explored mainly on nanoparticles functionalization for cancer‐related approaches.^[^ ^12^, ^13^ ^]^

We hypothesize that a bulk biomaterial functionalized with mannose can specifically and directly target MΦ within the local innate immune system at the implantation site. This targeted approach can modulate the implant‐related inflammatory response, thereby optimizing the biocompatibility and performance of the implant. Our previous research has shown that active signals on biomaterial surfaces effectively promote local retention of anti‐inflammatory MΦ (MΦ2) at the implantation site, significantly reducing the pro‐inflammatory milieu and properly initiating the healing cascade.^[^ ^4^ ^]^ Notably, collagen scaffolds functionalized with polysaccharides demonstrated that despite having similar mechanical and physical properties, sugars on the collagen surface enhance MΦ recruitment, resulting in improved scaffold integration within the tissue.^[^ ^14^ ^]^

MΦs show high plasticity and a dual role in the regenerative process.^[^ ^15^ ^]^ They adapt their phenotype in response to local signals and can be broadly classified as classically activated pro‐inflammatory (MΦ1) or alternatively activated anti‐inflammatory macrophages (MΦ2).^[^ ^16^ ^]^ MΦ polarization to the anti‐inflammatory phenotype is essential for progressing from the inflammatory phase to the proliferative and remodeling phases of wound healing. Indeed, chronic (nonhealing) wounds exhibit an increased number of MΦ1, which produce cytokines that hinder tissue repair.^[^ ^17^ ^]^ Early attempts at treating nonhealing wounds with macrophage‐based cell therapies have shown promising biological effects, including reductions in mortality and hospitalization time.^[^ ^18^ ^]^

Building on this rationale, our study introduces a robust method to fabricate collagen‐based patches (COL) functionalized with mannose, designed to enhance MΦ2 presence at the implant site. This is achieved by promoting MΦ recruitment at the implantation site and driving MΦ1‐to‐MΦ2 polarization. We termed this innovation Local Immunoactive Patch (LIP).  LIP was fabricated by either absorbing or crosslinking mannose onto collagen patches, which were comprehensively characterized for their chemical‐physical attributes. Furthermore, we explored LIP's immunomodulatory effect in vitro using bone marrow‐derived MΦ and assessed its efficacy in inhibiting pathogen growth. Finally, LIP was subcutaneously implanted in mice to analyze the molecular and cellular cascade activated by LIP in vivo. A direct comparison was made with COL to underscore the unique benefits of LIP in activating an alternative immunometabolism at the site of implant.

## 2 Materials and Methods

### 2.1 Patch Synthesis

#### 2.1.1 Collagen Patch Preparation

Type I collagen mesh from bovine tendon was prepared using a solvent‐casting method, as described previously.^[^ ^4^ ^]^ Briefly, 1 g of type I collagen (Viscofan Inc.) was dissolved in an acetate buffer (pH 3.5) to achieve a final concentration of 20 mg ml^−1^. The collagen suspension was then precipitated by adding a sodium hydroxide solution, adjusting the pH to 5.5. The resulting collagen slurry was mildly crosslinked with a 1.5 mM solution of 1,4‐butanediol diglycidyl ether (Sigma‐Aldrich) for 48 h. After crosslinking, the collagen was washed three times with water. The slurry was then molded in metal racks to a thickness of 2 mm and air‐dried under a fume hood for 3 days, resulting in a final thickness of 0.1 mm.

#### 2.1.2 Covalent Functionalization with Mannose (LIP)

COL were covalently functionalized with mannosamine hydrochloride by incubating them in a solution containing 0.1 M mannosamine hydrochloride and 0.2 mM glutaraldehyde as a crosslinker. This reaction was allowed to proceed for 24 h, followed by three washes with phosphate‐buffered saline (PBS). For the stability study, COL were incubated with the same concentration of mannosamine hydrochloride in the absence of glutaraldehyde (ABS).

### 2.2 Patch Characterization

#### 2.2.1 Fourier Transform Infrared (FTIR) Spectroscopy

FTIR spectra were recorded using a Nicolet 6700 FT‐IR Spectrometer (ThermoFisher Scientific) in attenuated total reflection mode. COL, ABS, and LIP patches were analyzed without any sample manipulation. Spectra were processed with EZ OMNIC software (Nicolet) following baseline correction between 1800 and 750 cm^−1^. The presence of mannose was further confirmed by histochemical staining using ELLA assay, which specifically labels proteoglycans.

#### 2.2.2 Contact Angle

The hydrophilicity/hydrophobicity of the resulting materials was assessed using a sessile drop measurement. In this method, a water droplet was placed on the surface of the patches, and the static contact angle was determined. The Young‐Laplace equation was fitted around the droplet using built‐in software provided by Biolin Scientific to define the contact angle.

#### 2.2.3 Patch Swelling

To evaluate the ability of the patch to absorb PBS, the lyophilized implant was weighed (Wd) and incubated under physiological‐like conditions for up to 1 month. At different time points, the hydrated patch was removed from the PBS, allowed to hang until no dripping water was observed, and weighed again (Wh). The percentage of absorbed PBS within the scaffold, defined as swelling, was calculated using the following equation: (swelling %) = [(Wh – Wd)/wd] × 100.

#### 2.2.4 Enzyme‐Linked Lectin Assay (ELLA)

To evaluate the stability of the binding between the different materials, COL, LIP, and ABS meshes were incubated in PBS for 21 days at 37 °C. At each time point, the samples were treated with 100 µl of 2% BSA in PBS and shaken for 14 h at 5 °C. Following the blocking step, the patches were incubated at room temperature with a 0.01 mg ml^−1^ solution of WGA fluorescently labeled (Sigma‐Aldrich) in PBS for 2 h shaking. After three washes in PBS, the patches were allowed to dry, and fluorescence was measured semi‐quantitatively by analyzing the fluorescent images using a confocal microscope. Normalized fluorescence intensity units were used to quantify the mannose onto the patches. Images are reported in 2D and 3D reconstruction. This analysis was conducted on four different patches.

### 2.3 Bone Marrow‐Derived Macrophage Isolation and Culture

Primary MΦ were isolated from murine bone marrow following established protocols.^[^ ^19^, ^20^ ^]^ After sacrificing C57BL/6 mice, surrounding tissues were removed from the femurs, and bones were cut at both ends. The bone cavity was flushed with complete media, using a 5‐mL syringe and a 25‐gauge needle. Bone marrow cells were mechanically separated into single‐cell suspensions, filtered using a 70‐µm strain, and plated in standard media constituted of High Glucose–Dulbecco's Modified Eagle Medium (HG‐DMEM) supplemented with 10% fetal bovine serum (FBS, v/v), and 1% penicillin (100 UI ml^−1^) streptomycin (100 mg ml^−1^), 0.25 mg ml^−1^ amphotericin B (v/v), and supplemented with macrophage colony‐stimulating factor (M‐CSF, 10 ng ml^−1^). Once obtained, 3 × 10^6^ MΦ were seeded onto COL, ABS or LIP patches in a concentrated drop of 30 µL and allowed to adhere for 20 min before media was added. Patches had previously been assembled in 12‐well plates using custom‐made inserts. Cultures were established in standard media at 37 °C in a humidified atmosphere with 5% CO_2_.

### 2.4 Effect of Mannose‐Functionalized Patches on Primary MΦ In Vitro

MΦ were cultured onto COL, ABS, and LIP patches for short time points (2, 4, 6, and 24 h) to assess their immediate response to the material. For comparison, 2D control groups were established, including untreated MΦ (control), and MΦ inflamed with pro‐inflammatory (lipopolysaccharide, LPS,100 ng ml^−1^) or anti‐inflammatory (Interleukin‐4, IL‐4, 20ng ml^−1^) molecules to induce the MΦ1 and MΦ2 phenotype, respectively. At each time point, cells were processed to detect phenotypical changes induced by exposure to the material, including morphology, expression of the mannose receptor (*Mrc‐1*), and MΦ1/MΦ2‐associated markers and proteins, as follows.

#### 2.4.1 Gene Expression Analysis

Gene expression was performed in triplicate from independent cultures (*n* = 3). Total RNA was extracted with Trizol reagent (Invitrogen) and subsequently purified with the RNeasy Mini Kit (Qiagen) to remove genomic DNA, proteins, and other organic contaminants. RNA concentration and quality were evaluated using the NanoDrop ND‐2000 spectrophotometer (NanoDrop Technologies). cDNA synthesis was carried out using the RT2 First Strand Kit (Qiagen), following the manufacturer's protocol. The resulting cDNA was analyzed using a commercial master mix and specific target probes for quantification to detect expression of pro‐inflammatory (*iNos*: Mm00440502_m1, *Tnf‐α*: Mm00443260_g1, *Il12‐β*: Mm00434174_m1) and anti‐inflammatory (*Mrc1*: Mm 00485148_m1, *Il10*: Mm 00439614_m1, *Arg1*: Mm 00475988_m1, *Tgf‐β1*: Mm01178820_m1) markers on an ABI StepOne plus Detection System (Applied Biosystems). Expression levels were normalized against reference gene (*Gapdh*: Mm99999915_g1). Positive control groups include MΦ cultured in the presence of IL‐4 to induce an MΦ2 phenotype or LPS for the MΦ1 phenotype.

#### 2.4.2 Enzyme‐Linked Immunosorbent Assay (ELISA)

ELISA was performed on supernatants from murine MΦ seeded onto adsorbed and crosslinked mannose patches (*n* = 3) using kits for mouse‐specific transforming growth factor‐beta (TGF‐β) (R&D Systems, Inc.) according to the manufacturer's instructions.

#### 2.4.3 Confocal Microscopy

Confocal microscopy was used to identify the presence of mannose receptor (MRC‐1) secondary to exposure to ABS or LIP compared to COL after 24 h. Samples were prepared by fixing cells in 4% paraformaldehyde for 20 min, labeling with Alexafluor488 Phalloidin (green), DAPI (blue) and MRC‐1 (red), before imaged using NIS‐Elements software (Nikon).

#### 2.4.4 Scanning Electron Microscopy (SEM)

MΦ adhesion onto LIP patches was assessed by SEM after 24 h. Samples were fixed at 4 °C overnight in a buffer solution containing 2.5% glutaraldehyde and 1% paraformaldehyde in PBS (pH 7.4) to preserve structural integrity. A sequential ethanol dehydration process was employed, exposing samples to a graded series of ethanol concentrations (25%, 50%, 70%, 90%, and 100%) for 10 min each, ensuring thorough dehydration. After mounting onto metal stubs, specimens were stored in a vacuum desiccator for 48 h to stabilize. For high‐resolution SEM imaging, samples were sputter‐coated with a 7 nm layer of Pt/Pd using the Plasma Sciences CrC‐150 Sputtering System (Torr International, Inc) and visualized at an accelerating voltage of 10 kV using the FEI Quanta 400 ESEM FEG.

### 2.5 LIP Antimicrobial Potential

Antimicrobial disc diffusion susceptibility testing was conducted using collagen disks (6‐mm diameter), both with and without cross‐linked mannose, against various bacterial strains. These included methicillin‐resistant *Staphylococcus aureus* (MRSA, *n* = 13), methicillin‐susceptible *S. aureus* with Bla Type A (MSSA, *n* = 12), MSSA with Bla Type C (*n* = 5), *Enterococcus faecalis* (*n* = 2), *Enterococcus faecium* (*n* = 3), and one strain each of *Pseudomonas aeruginosa* (strain PA14) and *Klebsiella pneumoniae*. The disk diffusion tests were carried out according to CLSI guidelines using Mueller–Hinton (MH) agar plates (BBL Becton Dickinson, MD).^[^ ^21^ ^]^ In brief, MH II agar plates were swabbed with bacterial cell suspensions adjusted to a 0.5 McFarland standard (1–2 × 10^8^ CFU ml^−1^), and the collagen disks, with or without cross‐linked mannose, were placed on the inoculated plates. After incubation at 37 °C overnight, the diameters (in mm) of the inhibition zones were measured and recorded.

### 2.6 In Vivo Studies

#### 2.6.1 Animals and Patch Subcutaneous Implantation

Animal studies adhered to approved protocols (AUP‐0115‐0002) set by the Institutional Animal Care and Use Committee (IACUC) at Houston Methodist Research Institute, following the standards outlined in the Animal Welfare Act and the Guide for the Care and Use of Laboratory Animals. Under sterile conditions and anesthesia induced by isoflurane inhalation, bilateral skin incisions were made along the dorsal area of each animal (left side designated for COL, right side for LIP). Balb/c mice (Charles River Laboratories) were used for material implantation. Animal studies were carried out using crosslinked mannose (LIP), which proved to be more stable overtime. Once the subcutaneous layer was accessed, 2 cm^2^ pockets were carefully prepared, and 1 cm × 1 cm COL and LIP patches were inserted into each pocket. At specified time points, animals were euthanized according to IACUC guidelines. An additional midline skin incision was made on the back where no material was implanted, serving as a control site.

#### 2.6.2 Bioluminescence

Bioluminescence images (BLI) were captured over 21 days using the Xenogen IVIS Imaging System (Caliper Life Sciences) and IVIS imaging software (Perkin Elmer, Santa Clara, CA).

#### 2.6.3 Tissue Isolation and Sample Preparation

At each designated time point, implanted materials along with surrounding tissues were retrieved and preserved in RNAlater® solution or lysis buffer to support downstream gene expression and proteomic analyses. For flow cytometry, specimens were digested with collagenase (Life Technologies) to isolate infiltrating cells for analysis.

### 2.7 Flow Cytometry

Flow cytometric analysis was performed to determine the number of cells infiltrating the patch overtime. To isolate cells, patches were digested with collagenase type I (2 mg ml^−1^ in Hank's Balanced Salt Solution containing calcium and magnesium, Life Technologies) for 30 minutes at 37 °C. The resulting cell suspensions were passed through a 70‐µm nylon mesh (BD Biosciences) to remove clumps and residual patch debris, centrifuged at 500 × *g* for 5 min, and subsequently fixed in 70% ethanol. To identify immune cell types within the patch at early time points (24, 48, and 72 h), cells were fixed, washed with FACS buffer (0.1% BSA), and labeled with antibodies against PE‐F4/80 (eBiosciences), APC‐CD206 (Biorbyt), and Pacific Blue‐CD86 (BioLegend). Control samples were incubated with isotype‐specific IgG or IgM to set the background signal. Each sample included a minimum of 20 000 events, analyzed using a BD LSR Fortessa™ cell analyzer (BD Biosciences), and data were processed in FlowJo software.

### 2.8 RT2 Profiler PCRs and Bioinformatic Analysis

RNA was isolated from explanted COL and LIP patches using Trizol reagent (Invitrogen) and then purified with the RNeasy Mini Kit (Qiagen) to remove genomic DNA, proteins, and organic contaminants. The concentration and quality of the RNA samples were verified using a NanoDrop ND‐2000 spectrophotometer (NanoDrop Technologies). Complementary DNA (cDNA) synthesis was conducted using the RT2 First Strand Kit (Qiagen), following the provided protocol. For comprehensive gene analysis, Mouse Inflammatory Cytokines & Receptors and Wound Healing RT2 Profiler PCR Arrays were employed on samples extracted at days 1 and 21, respectively. Real‐time PCR was performed using an ABI 7500 Fast Sequence Detection System (Applied Biosystems) with a two‐step cycling program and SYBR Green RT2 qPCR Master Mix (Qiagen). Threshold cycle values were processed with the PCR Array Data Analysis Software (SABiosciences, v3.5). Analysis of the Mouse Inflammatory Cytokines & Receptors Array was performed by calculating gene expression in COL and LIP groups, using baseline threshold values represented by the untreated incision samples. LIP expression values were then normalized to the COL values for comparison. The Wound Healing RT2 Profiler PCR Arrays were performed by comparing levels of expression between COL and LIP. In both cases, genes with a fold change (FC) > 1 were considered upregulated, while genes with FC < 1 were considered downregulated. The fold change values were then log2‐transformed to normalize the data and facilitate comparison of both upregulated and downregulated genes. For upregulated genes, log2(FC) values were positive, while downregulated genes exhibited negative values. To determine the statistical significance of the observed gene expression changes, *p*‐values were computed for each gene using an unpaired two‐tailed *t*‐test. To account for multiple comparisons and control the false discovery rate, the *p*‐values were adjusted using the Benjamini–Hochberg correction. The adjusted *p*‐values (q‐values) were used to assess the significance of differential expression between the COL and LIP groups. Genes with an adjusted *p*‐value less than 0.05 were considered statistically significant, indicating a meaningful change in expression between experimental groups, and used for gene ontology analysis. Pathway analysis was performed using NIH's DAVID tool to identify enriched biological pathways, with statistical significance determined based on false discovery rate (FDR) corrections. Visualization of the enriched Gene Ontology (GO) terms or gene sets was achieved using Enrichr.

### 2.9 Cytokine Proteome Profiling

The Mouse Cytokine Array Panel A (R&D Systems, catalog ARY006) was used to profile chemokines and cytokines secreted from COL and LIP patches at 1, 3, and 7 days postimplantation, enabling the detection of 29 distinct molecules. To collect adsorbed proteins, the scaffolds were cut into small fragments and suspended in cold RIPA buffer (Thermo Fisher Scientific) containing protease and phosphatase inhibitors. Protein extraction was achieved by sonicating the samples, followed by quantification using the Bradford assay (Bio‐Rad). For each sample, 40 µg of protein was processed according to the manufacturer's protocol. Protein array images were scanned, and pixel densities were quantified with the Protein Array Analyzer in ImageJ.^[^ ^22^ ^]^ Each array was quantified individually, using positive and negative control spots as maximum and minimum values. Mean values per cytokine were calculated considering technical replicates. To normalize to the normal expression of the control, control values for each cytokine (from INC group) were subtracted from values obtained from explanted COL and LIP patches. Relative expression populates a heatmap showing pixel density on a scale of 0 to 1, with 1 being the maximum intensity.

### 2.10 Statistical Methods

Statistical analysis was conducted using GraphPad Instat 3.00 for Windows (GraphPad Software, La Jolla, CA, USA). Each experiment was carried out in triplicate, and data are presented as the mean ± standard deviation. Significance was defined at a *p*‐value of ≤ 0.05, with *p*‐values < 0.01 considered highly significant. For comparing multiple groups, a one‐way ANOVA was applied, followed by the Student–Newman–Keuls post hoc test for detailed comparisons. An unpaired *t*‐test was performed to evaluate the significance of bacterial inhibition zones (mm) between LIP disks and control (no mannose) disks. For statistical analysis and graphical representation, inhibition zones measuring 0 mm were assigned a value of 1 mm.

## 3 Results

### 3.1 Covalent Linkage with Mannose Significantly Enhances Distribution and Functionalization Efficiency

**Figure 1:**
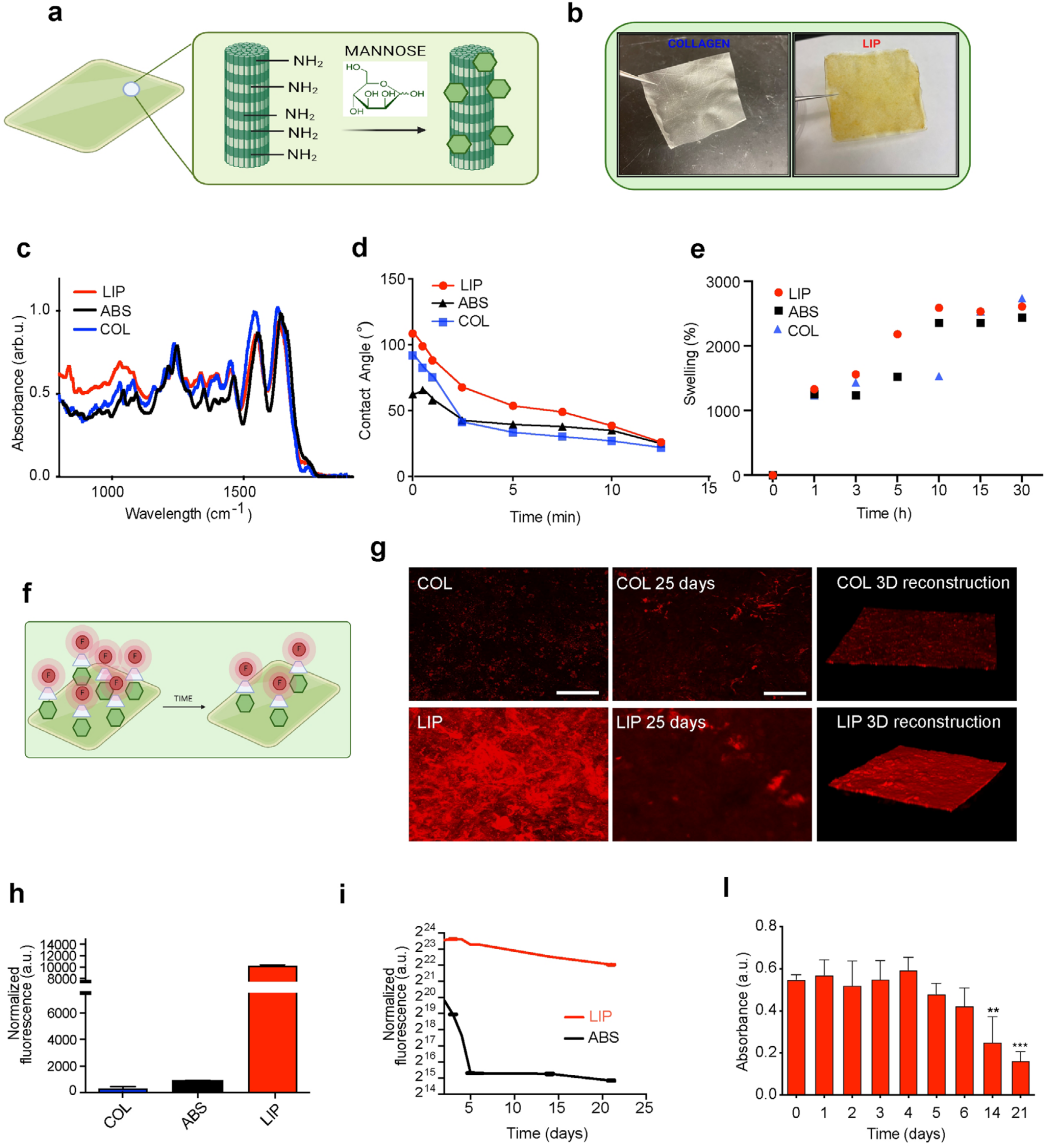
Synthesis and characterization of mannose‐functionalized collagen meshes. a) Schematic illustrating the chemical conjugation of mannose to collagen fibers. b) Visual comparison of unmodified collagen (COL) and mannose‐functionalized collagen (LIP), with LIP appearing opaquer, indicating successful mannose integration. c) FTIR spectra showing characteristic peaks between 1000 and 1300 cm⁻¹ for mannose in LIP, COL and ABS samples. d) Contact angle measurements reveal increased hydrophilicity in LIP and ABS compared to COL, confirming a different surface interface. e) Swelling capacity assessments indicate similar water retention levels among patches. f) Schematic representation of how WGA‐detection correlates with mannose functionalization. g) Fluorescence microscopy images showing mannose distribution on LIP at day 0 and after 25 days, with 3D reconstruction confirming uniform mannose presence throughout the scaffold compared to COL. h) Covalent mannose linkage in LIP shows greater functionalization stability compared to absorbed mannose. i,l) Fluorescence intensity measurements over time indicate a decrease in mannose stability under physiological conditions, highlighting reduced mannose retention after 25 days. Data are presented as mean ± SD from 3–5 independent experiments. Statistical significance: *p* < 0.01.

The synthesis and characterization of LIP were thoroughly investigated, with the results summarized in **Figure** **1**. The schematic in Figure 1a illustrates the process of mannose conjugation to the collagen matrix, highlighting the chemical interaction between mannose and collagen fibers. Visual inspection (Figure 1b, top right) reveals that the LIP appears opaquer compared to the clear, translucent unmodified collagen mesh (COL), suggesting successful mannose integration. The FTIR spectra (Figure 1c) of the LIP samples display characteristic peaks between 1000 and 1300 cm⁻¹, which are associated with sugar functionalization of proteins, indicating the presence of mannose on the collagen membrane. These peaks are absent in the COL and ABS samples. The contact angle measurements (Figure 1d) show a higher level of hydrophilicity for LIP in comparison with COL, further corroborating the presence of a different interface on functionalized patches. The swelling capacity of the patches was assessed as previously reported.^[^ ^23^ ^]^ No significant differences were found between the analyzed patches; however, all patches exhibited a high level of water retention (Figure 1e). WGA affinity binding with mannose^[^ ^24^ ^]^ was used to characterize the distribution and stability of the mannose functionalization on the patch surface. Fluorescence microscopy images (Figure 1f, g) show the initial distribution of mannose on the LIP and after 25 days. The 3D reconstruction (Figure 1g) provides a detailed view of the mannose distribution on the LIP confirming the presence of mannose throughout the scaffold compared to bare collagen. Figure 1h demonstrates that the covalent linkage with mannose results in a higher distribution and efficiency of functionalization. With 0.1 M of mannosamine, the mannose content is higher than the absorbed patch, indicating higher stability of the functionalization. However, over time, the fluorescence intensity decreases, indicating a gradual loss of mannose from the patch (Figure 1i,l). This suggests that while initial mannose functionalization is successful, there is a decline in stability over an extended period if maintained in physiological conditions of temperature and humidity. In summary, results presented in Figure 1 demonstrate successful mannose functionalization of LIP evidenced by FTIR analysis, different surface hydrophobicity, increased swelling capacity, and indirect quantification by WGA‐mannose interaction. However, the binding stability of mannose decreases over time, as indicated by the reduction in fluorescence intensity. The 3D reconstruction further confirms the homogeneous distribution of mannose on the LIP.

### 3.2 Crosslinked Mannose Shows Increased Stability Compared to Its Absorbed Counterpart

**Figure 2:**
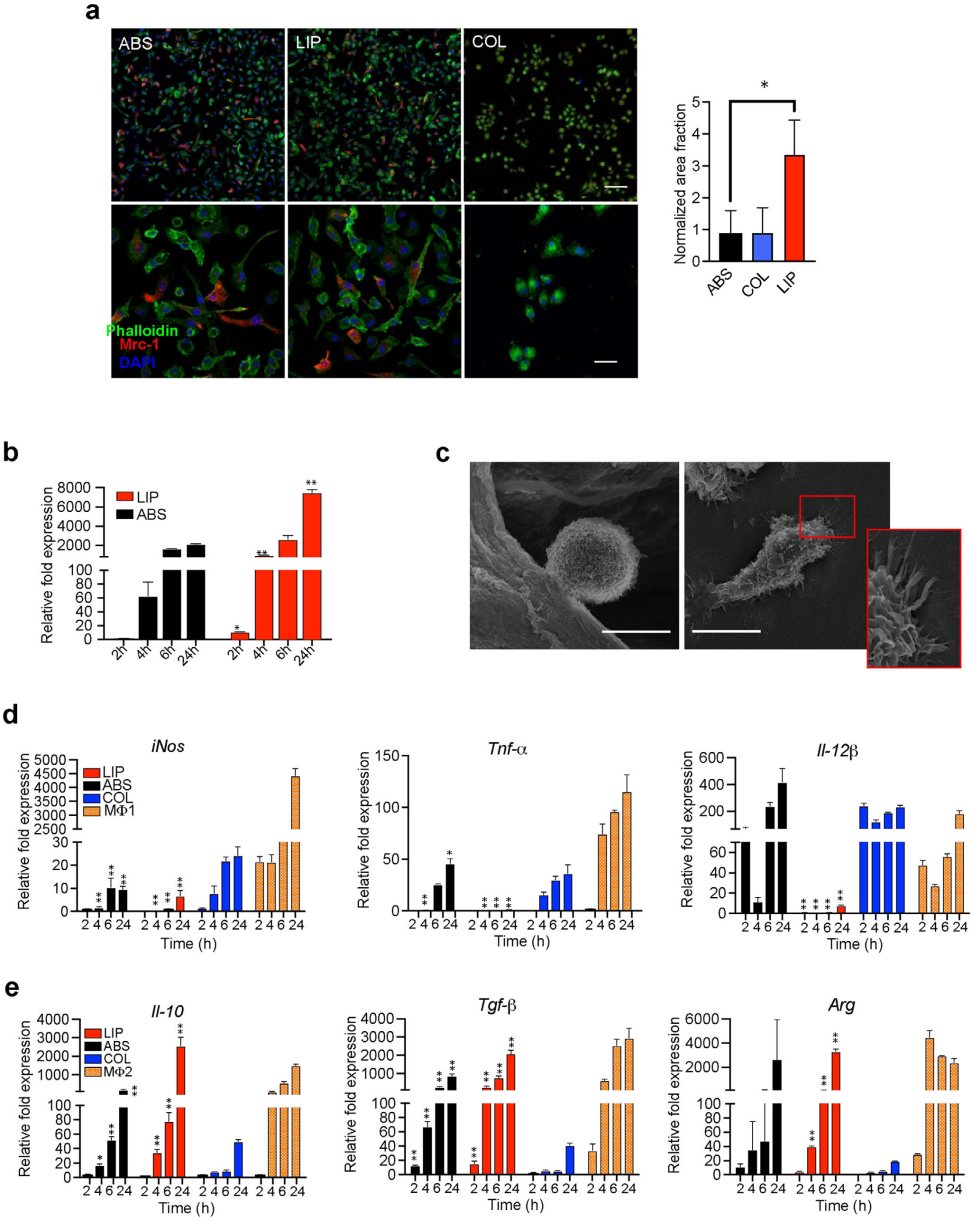
Comparison between absorbed and crosslinked mannose onto collagen patches in vitro. a) Representative confocal microscopy images at 4× (top) and 20× (bottom) magnification showing bone marrow‐derived MΦ grown onto COL, or collagen functionalized with mannose (ABS and LIP). Crosslinked mannose induced a more elongated morphology in MΦ and increases the expression of the mannose receptor (MRC‐1, red). Cells counterstained with phalloidin (green), DAPI (blue). Scale bars 10€µm. Graph showing quantification of MRC‐1 normalized to DAPI in LIP compared to COL and ABS show a significant increased expression levels in MΦ upon exposure to crosslinked mannose (*p* < 0.1). b) qPCR for the expression levels of *Mrc‐1* in MΦs exposed to ABS and LIP for 2, 4, 6, and 24 h. Data are presented as mean ± SD from 3 independent experiments. *Significant and **highly significant differences between ABS and LIP at *p* <  0.05 and *p* <  0.01, respectively. c) SEM images showing bone marrow‐derived MΦ adhere onto mannose functionalized collagen patches, showing a mix of rounded (left) and elongated (right) cells. Inset highlights the adhesion protrusions of MΦ in direct interaction with the surface. Gene expression analysis for pro‐ and anti‐inflammatory markers (d and e) on MΦ  grown onto ABS and LIP over time. Significant * and ** highly significant differences compared to COL at *p* < 0.05 and *p* < 0.01, respectively. Levels of expression induced by macrophage exposure to pro‐ or anti‐inflammatory cytokines are also shown as indicative of the MΦ1 and MΦ2 phenotype.

The first functional study was performed to assess the potential of LIP to increase the expression of the mannose receptor at a genetic and protein level on primary MΦ. Confocal microscopy confirmed that the presence of mannose (either ABS or LIP) on the collagen surface induces an increase in the expression of *Mrc‐1* on MΦ at a protein level at 24 h after seeding that is not observed when cells are cultured onto bare collagen patches (**Figure** **2**a). The expression of the *Mrc‐1* gene was monitored overtime, from 2 to 24 h. An increase in its expression was immediately noticed in the presence of crosslinked mannose (Figure 2b). While gene expression was sustained over 24 h in both groups, it was found to be more robust in LIP with values reaching over a threefold increase (7493 ± 324) compared to the absorbed condition (2105 ± 61). SEM analysis revealed high‐resolution images showing primary MΦ interacting with both types of patches. Qualitative observations suggest that the cells interact and adhere to both patches, however, the macrophage phenotype appears more elongated on LIP showing more adhesion phillopodia interaction, while rounder‐shaped MΦ are more prevalent on COL (Figure 2c).

### 3.3 Crosslinked Mannose Modulates Macrophage Polarization In Vitro

To assess the immediate reaction of MΦ to mannose‐functionalized patches and determine their immunomodulatory potential, primary cells were cultured onto patches and the expression of pro‐ and anti‐inflammatory genes was evaluated at 2, 4, 6, and 24 h compared to respective positive controls (MΦ1 or MΦ2) or COL. When pro‐inflammatory genes were analyzed, a significant reduction in their expression upon exposure to mannose‐functionalized patches compared to positive control was found (Figure 2d). No *Tnf‐a* expression was found in LIP at any time point, whereas in ABS an expression pattern similar to COL was observed. *iNos and Il‐12a* expression was only detected at the latest time point (24 h) in LIP with values that are still lower (6.8 ± 2.2‐fold) than other groups (24.31 ± 3.6‐ and 4432 ± 247‐fold for COL and MΦ1 grown in 2D, respectively), including ABS (9.56 ± 1.25‐fold). ABS seemed to also induce a pro‐inflammatory phenotype in primary MΦ to a certain extent compared to its crosslinked counterpart, with expression levels found to increase overtime and become greater than COL and positive control (419 ± 99‐fold vs 236 ± 9‐fold and 186 ± 23‐fold, respectively) at 24 h for *Il‐12b*. A less stable expression pattern over time was found in the ABS compared to LIP. A time‐dependent rise in the expression of anti‐inflammatory genes (*Arg*, *Il‐10*, and *Tgf‐β*) was noted in response to mannose, both in its absorbed and crosslinked forms. In particular, LIP showed a 3314 ± 192‐fold, 2088 ± 194‐fold, and 2841  ±  468‐fold increase in *Arg*, *Tgf‐β* and *Il‐10* expression, respectively. *Arg* and *Il‐10* expression values were even greater than those induced by MΦ exposure to anti‐inflammatory cytokines, assessed around 2405 ± 326 and 1721 ± 84‐fold, respectively. Comparable expression levels were identified for the third gene tested, *Tgf‐β* (Figure 2e). Quantification of TGF‐*β* released by MΦs secondary to the exposure to mannose revealed comparable levels between ABS and LIP at 24 h (2.98 ± 0.58 and 3.12 ± 0.28, respectively) (Supplementary Figure S1, Supporting Information). Similarly, we observed a highly variable intermittent expression pattern of TGF‐β over time in the ABS patch compared to its crosslinked counterpart. Due to the variability of the absorbed functionalized patch, we focused exclusively on LIP and its control, the bare collagen (COL) patch, for the experimental phase.

### 3.4 LIP Displays an Inhibitory Effect on Bacterial Growth

**Figure 3:**
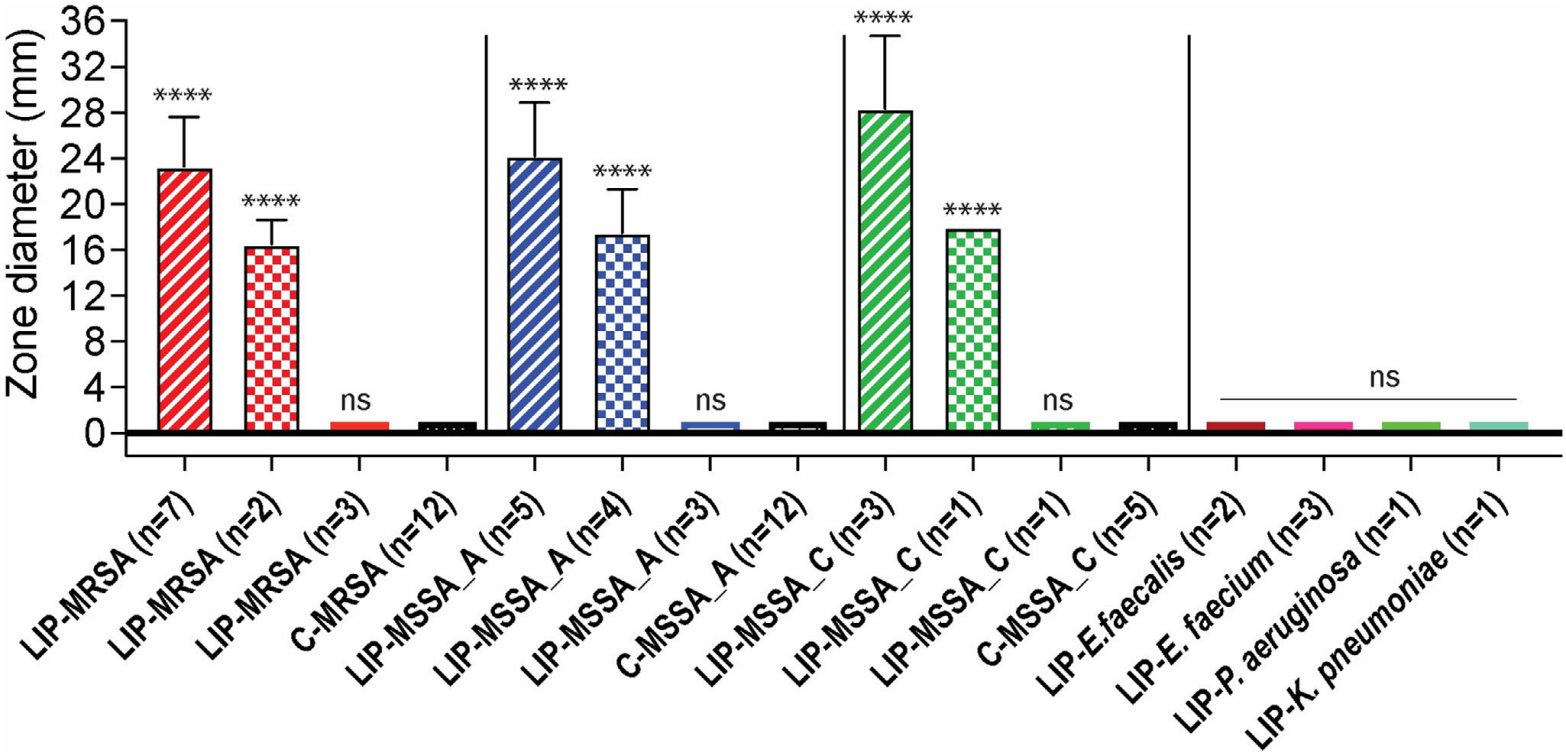
Antimicrobial potential of LIP compared to COL. The graph compares the antimicrobial activity of LIP disks and COL disks. Statistical significance was determined using an unpaired *t*‐test (**** = *p* < 0.0001; ns = not significant). The number of replicates per bacterial strain is indicated in the graph. Values of 0 mm were adjusted to 1 mm for visualization on the graph and for statistical analysis.

Given the potential application of this patch for wound healing, its relationship with infections is critical. To address this, we conducted a proof‐of‐concept series of tests to determine whether mannose integration on the patch confers bactericidal or bacteriostatic properties. LIP demonstrates a 75–80% inhibition zone against *S. aureus*, regardless of MRSA or MSSA (**Table** **1**, **Figure** **3**). Among the 13 *S. aureus* (MRSA) isolates, LIP disks exhibited growth inhibition zones of ≥20 mm in 7 (54%) and between 12 and 20 mm in 2 (15%) of the strains. No inhibition zones were observed in 4 (31%) of the MRSA isolates (Supplementary Figure S2a). Among the 12 *S. aureus* (MSSA, Bla type A) isolates, 5 (42%) showed inhibition zones of ≥20 mm, and 4 (33%) showed inhibition zones between 12 and 20 mm (Figure S2b, Supporting Information). No inhibition zones were observed in 3 (25%) of these MSSA (Bla type A) isolates. For the 5 *S. aureus* (MSSA, Bla type C) isolates, 3 (60%) displayed inhibition zones of ≥20 mm, and 1 (20%) displayed a zone between 12 and 20 mm. No inhibition zone was observed in 1 (20%) of the MSSA (Bla type C) isolate. LIP disks showed no inhibitory effect against *P. aeruginosa*, or *K. pneumoniae* strains (Figure S2c, Supporting Information), nor against *E. faecalis* and *E. faecium* (Figure S2d, Supporting Information). Control disks containing collagen alone did not show any inhibition zones against the test bacteria.  A graph summarizing the inhibition zone diameters is reported in Figure 3 to show statistical significance.

**Table 1:** Inhibition of bacterial growth (in mm) by LIP compared to collagen patches.

Bacteria used	Zone diameter (mm)^*)^	
	LIP	COLLAGEN
***Methicillin‐resistant S. aureus*** **(*n* = 13)**		
MRSA (*n* = 7)	≥20 mm	0 mm
MRSA (*n* = 2)	12–20 mm	0 mm
MRSA (*n* = 4)	0 mm	0 mm
**Methicillin‐susceptible *S. aureus*** **(*n* = 17)**		
MSSA Bla Type A (*n* = 5)	≥20 mm	0 mm
MSSA Bla Type A (*n* = 4)	12–20 mm	0 mm
MSSA Bla Type A (*n* = 3)	0 mm	0 mm
MSSA Bla Type C (*n* = 3)	≥20 mm	0 mm
MSSA Bla Type C (*n* = 1)	12–20 mm	0 mm
MSSA Bla Type C (*n* = 1)	0 mm	0 mm
***E. faecalis* (*n* = 2)**	0 mm	0 mm
***E. faecium* (*n* = 3)**	0 mm	0 mm
***P. aeruginosa* (*n* = 1)**	0 mm	0 mm
***K. pneumoniae* (*n* = 1)**	0 mm	0 mm

*) **=** Zone diameter (mm) includes 8 mm of disk diameter.

### 3.5 LIP Reduces Inflammation at the Site of Implant in a Subcutaneous Mouse Model

**Figure 4:**
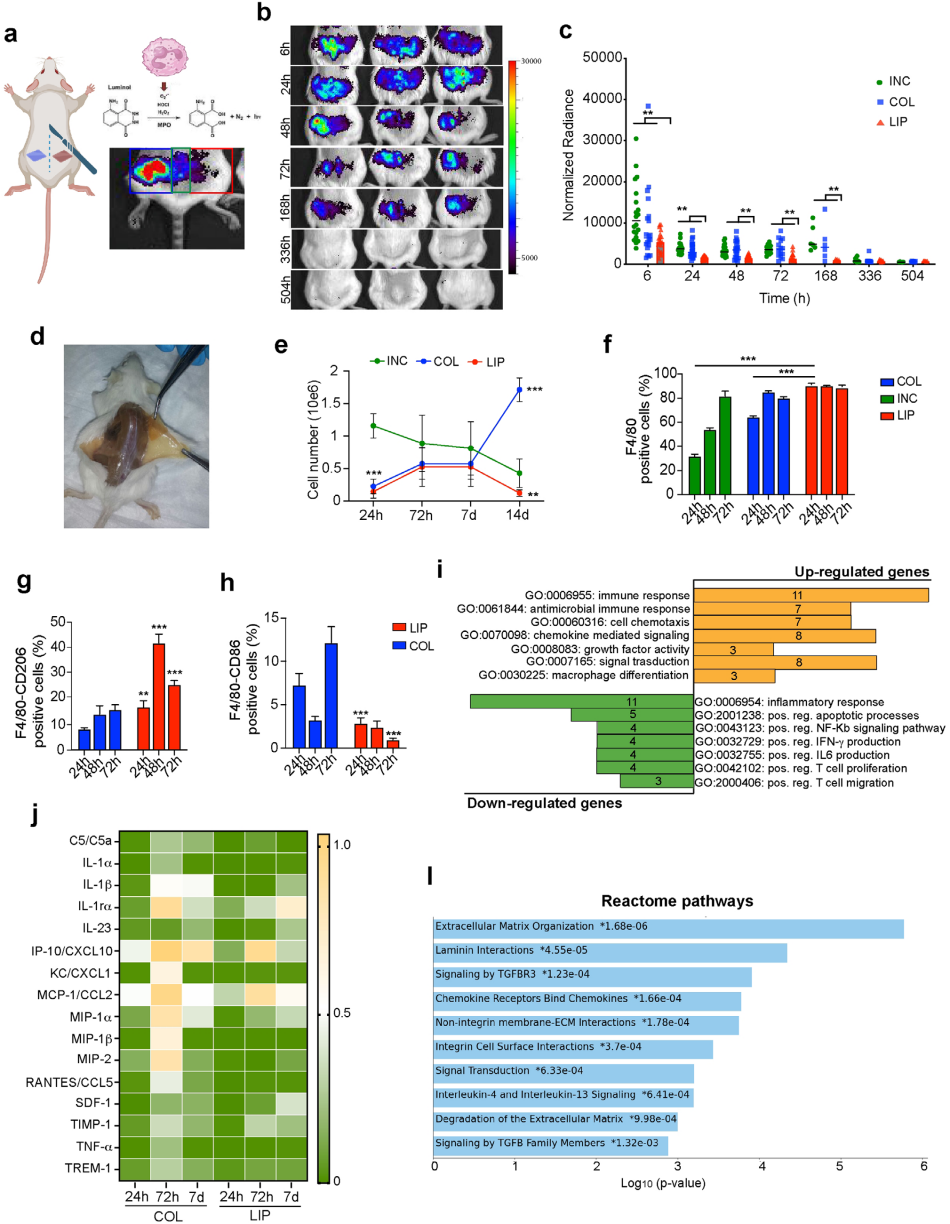
Immunomodulatory effect of LIP upon subcutaneous implantation. a) Schematic representation of the surgery. LIP (right) and COL (left) were implanted subcutaneously, while incision was performed at the midline level (INC). b) Representative images of bioluminescence in mice at different time points following subcutaneous implantation. Color scale represents radiance (p/sec/cm^2^/sr]. c) Mice were imaged at different time points (6, 24, 48, and 72 h, and 7, 14, and 21 days). Graph shows quantification of the intensity of the bioluminescent signal overtime, corresponding to the recruitment of immune cells at the site of implant/incision (*n* = 8). Signal associated to the midline incision is also shown. d) Representative image of the subcutaneously implanted patch during tissue explant. e) Number of cells harvested from explanted LIP or COL at early (24 and 72 h) and late (7 day) time points. Data are presented as mean ± SD from 3 independent experiments. f) Quantification of F4/80^+^ cells recruited at the site of implant (LIP and COL) as performed by flow cytometric analysis at 24, 48, and 72 h. MΦ infiltration in tissues upon incision (INC) with no implant is included as reference. Percentage of anti(F4/80^+^/CD206^+^)‐ and pro(F4/80^+^/CD86^+^) MΦ overtime (f and h, respectively). Data are presented as mean ± SD from 3 independent experiments. Significant ** and *** highly significant differences compared to COL at *p* < 0.01 and *p* < 0.001, respectively. i) GO analysis of genes found up‐ and down‐regulated in LIP compared to COL 1 day after implantation, as resulted from inflammatory cytokines and receptors array performed by DAVID, highlighting enriched biological processes. Significant GO terms were selected based on FDR< 0.05. Data were normalized against baseline and fold increase determined in LIP compared to COL. j) Heatmap of protein relative expression levels found in COL and LIP explants over time, as assessed by Proteome Profiler Array with intensity values normalized across samples. l) Bar chart highlighting top enriched biological processes using Enrichr on differentially upregulated genes in LIP compared to COL as determined by wound healing array 21 days postimplant. Enriched pathways are ranked based on the strength of their association with the input gene list.

LIP patches were implanted subcutaneously in mice to monitor their immunotuning potential over time at a cellular and molecular level. We performed noninvasive BLI to assess the inflammatory response related to the implant^[^ ^23^ ^]^ via luminol, a small molecule that reacts with myeloperoxidase enzyme expressed by activated innate immune cells.^[^ ^25^ ^]^ A schematic of the implant design is shown in **Figure** **4**a, with LIP and COL patches implanted subcutaneously in mice and a midline incision performed as a baseline. Images of the bioluminescent signal associated with each implant overtime (6–504 h postimplant) (Figure 4b) show a significant reduction (*p* < 0.01) in the signal associated to the LIP implant (right side) compared to COL (left side) and midline incision as soon as 6 h, with an overtime decay that reaches the lowest point after 7 days (sixfold decrease compared to other groups) (Figure 4c). To better understand the molecular mechanisms activated by the material, explants were analyzed to determine the extent of cell recruitment at early (1 and 3 days) and long (7 and 14 days) time points. A representative image of the subcutaneously implanted material after 21 days is shown in Figure 4d. One day after implantation both LIP and COL were colonized by a layer of infiltrating cells, not significantly different in number, as revealed by flow cytometry (Figure 4e). The number of recruited/proliferating cells, however, increased overtime in COL reaching the highest peak at 14 days postimplantation (1 716 000 ± 181 710 cells). On the other hand, it returned to baseline in the LIP group (124 500 ± 50 230 cells), with a trend that was found comparable to the untreated incision (429 000 ±  217 736 cells) although displaying a less crowded (3.5‐fold) cellular landscape. Among recruited cells, immune cell types were assessed at early time points. Significantly higher levels of F4/80 positive cells were found in LIP compared to COL as early as 24 h postimplantation (Figure 4f). A deeper evaluation revealed a higher percentage of MΦ in LIP (90% vs 68% in COL), with a twofold increase in MΦ2 compared to COL (Figure 4g). This increase was four times higher at 48 h and then decreased, though it remained sustained three days postimplantation. Concomitantly, the percentage of pro‐inflammatory MΦ (CD86+) was significantly lower in LIP compared to COL (Figure 4h), never exceeding 3%. The gating strategy used to identify CD206+ or CD86+ cells within the F4/80‐positive population, along with individual plots illustrating differences in positive cell infiltrates in COL and LIP over time, is shown in Supplementary Figure S3 (Supporting Information) and data are displayed in **Table** **2**.

**Table 2:** Percentage of infiltrating cells in LIP, COL, and INC at early time points. Flow cytometric analysis revealing the percentage of infiltrating cells in LIP, COL, and at the incision site (INC) at various time points. Cells were stained for the macrophage marker F4/80 and for CD206 and CD86 to identify M2‐like and M1‐like macrophage populations, respectively. Data are presented as mean ± standard deviation (*n* = 3).

	F4/80 + cells	F4/80+‐CD206+ cells	F4/80+‐CD86+ cells
**LIP 24 h**	90.3 ± 1.12	16.86 ± 2.5	2.86 ± 0.63
**LIP 48 h**	90.13 ± 0.91	42.03 ± 3.53	2.38 ± 0.73
**LIP 72 h**	88.6 ± 1.31	25.7 ± 1.65	0.96 ± 0.18
**COL 24 h**	67.33 ± 1.52	8.17 ± 0.60	7.27 ± 1.36
**COL 48 h**	85.16 ± 1.12	14.1 ± 3.35	3.23 ± 0.40
**COL 72 h**	79.9 ± 0.70	15.9 ± 1.91	12.16 ± 1.83
**INC 24 h**	31.8 ± 1.81	1.48 ± 0.18	0.99 ± 0.13
**INC 48 h**	53.7 ± 1.76	6.75 ± 0.50	10.71 ± 7.73
**INC 72 h**	81.8 ± 0.47	6.23 ± 0.18	1.54 ± 0.24

### 3.6 LIP Orchestrates a Faster Healing Cascade In Vivo

The gene expression profile of cells collected from the patches 1‐day upon implantation was further explored by performing an Inflammatory Cytokines & Receptors array. Data indicates significant differences in expression patterns between LIP and the healing cascade activated by bare collagen (**Table** **3**). Gene ontology analysis identified that 20 of the genes downregulated in LIP are associated with a diminished inflammatory response. In contrast, 13 upregulated genes are linked to the regulation of immune response, macrophage differentiation, antimicrobial immune response, and cell chemotaxis (Figure 4i). The inflammatory profile at the implantation site was assessed using a Proteome Profiler Array at specific time points: days 1, 3, and 7 (Figure 4j, Supplementary Table S1, Supporting Information). Overall, higher cytokine expression was observed in the COL explants compared to LIP, with COL displaying the most pronounced inflammatory profile at 72 h postimplantation. The expression of interleukin‐1 receptor antagonist (IL‐1rα), an anti‐inflammatory cytokine, progressively increased in LIP explants, reaching peak intensity on day 7 postimplantation. In contrast, this trend was not seen in COL, where the highest IL‐1rα expression was recorded at 72 h and decreased by ≈50% by day 7. The pro‐inflammatory cytokine interleukin‐1 alpha (IL‐1α) was more highly expressed in COL explants at both the 72‐h and 7‐day time points compared to LIP explants, which exhibited baseline expression levels. Similarly, Macrophage Inflammatory Protein‐1 Alpha (MIP‐1α) was expressed at higher levels in COL explants than in LIP, peaking at 72 h postimplantation. Interestingly, the expression trend in LIP explants was distinct, showing no detectable MIP‐1α expression at 24 h but increasing to a peak on day 7, although still lower than that of COL explants. A high relative expression of the pro‐inflammatory Monocyte Chemoattractant Protein‐1 (MCP‐1) was observed in both explant types at all time points, with particularly high expression at 72 h postimplantation, which then declined by day 7. To validate these results, we examined the expression of 84 genes associated with wound healing and tissue regeneration at the 21‐day mark. Molecular changes induced in vivo by mannose were found in the upregulated expression of 7 genes (**Table** **4**), associated with biological processes related to tissue repair, including ECM reorganization, cell matrix adhesion, and tissue remodeling (Figure 4l).

**Table 3:** Differentially expressed genes in the 24‐h in vivo LIP implant, compared to COL, were analyzed using a PCR array focused on pro‐inflammatory cytokines and receptors. Statistical significance was determined by calculating adjusted *p*‐values using the Benjamini–Hochberg correction for multiple comparisons. Genes with adjusted *p*‐values <0.05 were considered significantly differentially expressed.

Symbol	Refseq	Description	Fold Change (log2)	Adj. *p*‐value
Down‐regulated genes				
*Ccl17*	NM_001252129	C‐C Motif Chemokine Ligand 17	−9.43	0.000766
*Ccl3*	NM_001305	C‐C Motif Chemokine Ligand 3	−9.4	0.037071
*Ccl4*	NM_001202907	C‐C Motif Chemokine Ligand 4	−10.49	0.01787
*Ccl5*	NM_001301159	C‐C Motif Chemokine Ligand 5	−8.86	0.005592
*Csf1*	NM_001301051	Colony Stimulating Factor 1	−7.03	0.005136
*Csf2*	NM_001301073	Colony Stimulating Factor 2	−4.13	0.01087
*Csf3*	NM_001301228	Colony Stimulating Factor 3	−7.20	0.039937
*Cxcl10*	NM_001564	C‐X‐C Motif Chemokine Ligand 10	−8.31	0.01087
*Ccr5*	NM_001295472	C‐C Motif Chemokine Receptor 5	−1.29	0.073752
*Ccr8*	NM_001295126	C‐C Motif Chemokine Receptor 8	−7.71	0.000766
*Fasl*	NM_010176	Fas Ligand, TNF Superfamily, 6	−10.50	0.005592
*Ifn‐g*	NM_000619	Interferon gamma	−0.59	0.028275
*Il1b*	NM_000576	Interleukin 1 beta	−8.75	0.047617
*Lta*	NM_000595	Lymphotoxin alpha	−5.14	0.047617
*Ltb*	NM_000596	Lymphotoxin beta	−10.35	0.01087
*Osm*	NM_002746	Oncostatin M	−7.16	0.018538
*Tnf‐a*	NM_000594	Tumor Necrosis Factor alpha	−8.32	0.048497
*Tnfrsf11b*	NM_003185	Tumor Necrosis Factor Receptor, 11b	−0.58	0.005592
*Tnfsf4*	NM_003992	Tumor Necrosis Factor Ligand, 4	−1.84	0.046018
*Vegfa*	NM_001025370	Vascular Endothelial Growth Factor alpha	−2.40	0.018538
**Up‐regulated genes**				
*Bmp2*	NM_001200	Bone Morphogenetic Protein 2	4.36	0.028275
*Ccl1*	NM_009804	C‐C Motif Chemokine Ligand 1	11.07	0.005592
*Ccl11*	NM_009140	C‐C Motif Chemokine Ligand 11	3.88	0.020051
*Ccl19*	NM_009927	C‐C Motif Chemokine Ligand 19	1.37	0.000766
*Ccl6*	NM_009141	C‐C Motif Chemokine Ligand 6	2.74	0.037071
*Ccl8*	NM_009145	C‐C Motif Chemokine Ligand 8	5.57	0.015596
*Ccl9*	NM_009146	C‐C Motif Chemokine Ligand 9	4.54	0.039423
*Ccr6*	NM_009919	C‐C Motif Chemokine Receptor 6	19.71	0.006998
*Cxcl12*	NM_001025	C‐X‐C Motif Chemokine Ligand 12	5.87	0.005592
*Il17b*	NM_008367	Interleukin 17 beta	2.41	0.018538
*Il7*	NM_010550	Interleukin 7	3.00	0.035678
*Tnfsf10*	NM_011640	Tumor Necrosis Factor Ligand, 10	2.39	0.018538
*Tnfsf13*	NM_133563	Tumor Necrosis Factor Ligand, 13	1.97	0.048784

**Table 4:** Under‐ and over‐expressed genes in 21‐day LIP in vivo implant, compared with COL, profiled on wound healing PCR array. Statistical significance was determined by calculating adjusted *p*‐values using the Benjamini–Hochberg correction for multiple comparisons. Genes with adjusted *p*‐values <0.05 were considered significantly differentially expressed.

	Refseq	Description	Fold Change(log2)	Adj. *p*‐value
Up‐regulated genes				
*Cd40lg*	NM_001269092.1	CD40 ligand	5.07	0.004549
*Col4a1*	NM_001845.4	Collagen, type IV, alpha 1	9.74	0.007499
*Cxcl11*	NM_001167513.2	C‐X‐C motif chemokine ligand 11	5.93	0.003008
*Cxcl5*	NM_011907.3	C‐X‐C motif chemokine ligand 3	3.82	0.003008
*Fgf2*	NM_008006.3	Fibroblast growth factor 2	5.90	0.045349
*Itga2*	NM_008396	Integrin alpha 2	13.74	0.0003
*Timp1*	NM_011593	Tissue inhibitor of metalloproteinase 1	0.49	0.037694
**Down‐regulated genes**				
*Cdh1*	NM_009859.3	E‐Cadherin	−12.15	0.000117
*Csf2*	NM_009977.3	Colony stimulating factor 2	−7.12	0.045726
*Ifng*	NM_010510.3	Interferon Gamma	−9.54	0.000003
*Tgf‐a*	NM_011577.2	Transforming Growth Factor Alpha	−3.59	0.010199

## 4 Discussion

This paper introduces an innovative patch designed to target innate immunity at the site of the implant by targeting MΦ, thereby promoting the localized secretion of potent biological factors. To this, mannose has been exploited as a key molecule to facilitate macrophage recruitment and induce transient expression of the mannose receptor (*Mrc‐1*), ultimately driving tissue‐repair functions. Several groups, including us,^[^ ^4^ ^]^ have previously demonstrated transient modulation of key molecules and ligands as an effective strategy to enhance cell migration and adhesion, potentially leading to improved therapeutic outcomes when applied in vivo.^[^ ^26^, ^27^, ^28^ ^]^ Moreover, mannose is a natural inhibitor of glycolysis in immune cells, which has been shown as a potential strategy for regeneration.^[^ ^29^ ^]^ Furthermore, its well‐documented antibacterial properties, commonly utilized in the prevention and treatment of UTIs,^[^ ^10^, ^30^ ^]^ have been harnessed to introduce an additional layer of control, which could be advantageous for potential applications in reconstructive surgeries. The integration of mannose within the collagen patches offers a promising platform for accelerating tissue regeneration by activating the natural immune response without supporting bacterial adhesion and growth.

Collagen mannosylation was achieved through either noncovalent absorption or direct covalent functionalization. However, crosslinking mannose (LIP) proved essential for ensuring reliable surface exposure of the sugar and providing material stability for up to 21 days.^[^ ^4^, ^31^, ^32^ ^]^ The chemical stability of the materials was demonstrated through cellular and molecular assays using bone marrow‐derived MΦs, confirming that the presence of mannose on the patches induces higher *Mrc‐1* expression compared to bare collagen, particularly within the first 24 h. Notably, LIP resulted in a threefold increase in *Mrc‐1* expression compared to ABS, suggesting superior efficacy in modulating MΦ behavior. A similar trend emerged when evaluating the inflammatory phenotype of primary MΦ over time. Both absorbed and crosslinked mannose reduced the expression of pro‐inflammatory markers (*iNos*, *Tnf‐α*, and *Il‐12b)* while upregulating anti‐inflammatory markers (*Il‐10*, *Tgf‐β*, and *Arg*). This behavior parallels phenotypes observed in chemically induced MΦ1 and MΦ2 used as positive controls. Production of TGF‐β at a protein level further confirms this observation, being a pivotal factor in tissue repair, regulating inflammation, extracellular matrix production, and remodeling, as well as promoting cellular responses necessary for successful wound healing and regeneration.^[^ ^33^ ^]^ These findings support prior studies indicating that mannose functionalization enhances MΦ targeting^[^ ^13^ ^]^ and induces clustering of the mannose receptor,^[^ ^12^ ^]^ thereby promoting an anti‐inflammatory phenotype. The absorbed mannose group demonstrated a more unstable response over time, characterized by increased variability among replicates and diminished overall efficacy compared to its crosslinked mannose counterpart. This variability highlights the critical role of covalent linkage in ensuring consistent bioactivity and stability. Consequently, LIP was selected as the preferred strategy, providing a more reliable platform to assess both antibacterial efficacy and performance in in vivo models. The stability of mannose functionalization achieved through glutaraldehyde crosslinking is a significant strength of our platform. However, we recognize the inherent cytotoxicity of glutaraldehyde^[^ ^34^ ^]^ and its potential impact on biocompatibility. Alternative strategies, such as the use of genipin or carbodiimide‐based crosslinking chemistries,^[^ ^35^ ^]^ offer promising solutions to address this limitation. Future iterations of our platform will incorporate these alternatives to enhance safety while preserving the functional integrity of mannose.

The control of the foreign body reaction that we aim to achieve modulating the inflammatory response at an implant site must also consider the response of pathogens to the implanted material. Surgical site infections associated with implanted devices pose a significant burden on healthcare systems, making infection control a critical aspect of implant safety and effectiveness.^[^ ^36^, ^37^ ^]^ Several types of biomaterials have demonstrated potential to inhibit bacterial growth, primarily through intrinsic material properties (i.e., chitosan, graphene oxide, silver ions, zinc oxide) or via the incorporation of antimicrobial agents (i.e., antimicrobial peptides, iodine, dopamine).^[^ ^38^ ^]^ Previous efforts to functionalize mannose within biomaterials have also demonstrated its potent antibacterial and antibiofilm activities, particularly against multidrug‐resistant bacteria.^[^ ^39^, ^40^ ^]^ Our study not only corroborates these findings but also underscores the significant inhibitory effects of LIP, achieving up to 80% reduction in *S. aureus* growth, a pathogen frequently associated with wound infections. In our antimicrobial testing, the inhibition zone observed is primarily attributed to the direct interaction between bacteria and the mannose‐functionalized surface of the LIP disks. This suggests a bacteriostatic or bactericidal effect localized to the contact area, likely mediated through surface interactions such as the engagement of bacterial lectins by the covalently bound mannose. While the mannose is covalently linked to the collagen substrate, there may be a small degree of mannose release over time due to environmental factors or partial cleavage of bonds, contributing to the observed antibacterial effect. Notably, this antibacterial efficacy extended beyond methicillin‐sensitive strains to include some methicillin‐resistant strains, highlighting its potential application in bandage development, especially considering the prevalence of MRSA infections in wounds such as cuts or abrasions.^[^ ^41^ ^]^ Conversely, when LIP disks were exposed to other common pathogens, including *P. aeruginosa*, *K. pneumoniae*, *E. faecalis*, and *E. faecium*, no antibacterial effect was observed. This lack of effect suggests that the antibacterial activity may be selectively triggered by mannose in response to specific bacterial strains. The absence of any activity with bare collagen disks further highlights the unique role of mannose in mediating this selective antibacterial response, underscoring its critical contribution to the observed effects, and showing promise in limiting antibiotic‐resistant infections. We hypothesize that mannose functionalization may interfere with bacterial adhesion molecules, particularly MSCRAMMs (microbial surface components recognizing adhesive matrix molecules), which are critical for *S. aureus* attachment to host tissues and surfaces. By mimicking natural glycosylated ligands, mannose‐functionalized surfaces could competitively inhibit these adhesion processes, thereby impairing bacterial colonization and biofilm formation. Additionally, mannose may influence bacterial quorum sensing, a key regulatory mechanism governing group behavior in *S. aureus*, including biofilm development and virulence factor production. Mannose's structural similarity to natural carbohydrates could allow it to disrupt these signaling pathways, though further studies are required to validate this hypothesis. These potential mechanisms provide a foundation for follow‐up investigations aimed at elucidating the precise antibacterial effects of mannose‐functionalized materials and exploring their application in preventing *S. aureus* infections, particularly in implantable devices and wound care. This hypothesis is supported also by the selective antibacterial effect of LIP, with significant inhibition of *S. aureus* (MRSA and MSSA) but no activity against *P. aeruginosa*, underscores the specificity of mannose effect. We hypothesize that this selectivity arises from interactions between mannose and staphylococcal surface components, such as MSCRAMM. In contrast, *P. aeruginosa* possesses an outer membrane with lipopolysaccharides and efflux systems that may prevent effective interaction with mannose. Additionally, its quorum sensing and biofilm pathways are regulated by mechanisms distinct from those of *S. aureus*, potentially explaining the lack of antibacterial activity. Future studies will focus on elucidating these mechanisms to confirm the role of mannose in selective bacterial targeting.

The potential of LIP to modulate the immune response by directly acting over APC cells was evaluated through subcutaneous implantation in a murine model. This approach allowed for an in‐depth analysis of the temporal immune modulation and its impact on promoting a cascade toward tissue homeostasis. Bioluminescence analysis conducted over 21 days revealed a remarkable reduction in the inflammatory response triggered by LIP implantation, with inflammation barely detectable at early time points. This response significantly outperformed both bare collagen patches and the natural healing process observed following an incision. These findings emphasize the efficacy of LIP in attenuating inflammation and promoting a more conducive environment for wound healing, further supporting previously published studies that emphasize the pivotal role of glycosaminoglycans in fostering regenerative environments.^[^ ^4^, ^42^, ^43^ ^]^ Additional insights into the molecular and cellular mechanisms activated locally by LIP were gleaned from explanted tissues at various time points. Notably, few or no adhesions were observed when LIP was implanted, a stark contrast to the collagen counterpart. This aspect is particularly crucial for the development of biomaterials in tissue engineering applications, as it minimizes complications associated with postsurgical adhesion formation,^[^ ^44^, ^45^ ^]^ thereby improving patient outcomes and the overall success of regenerative medicine approaches.^[^ ^46^, ^47^ ^]^

The analysis of infiltrating cells showed no significant differences in the overall cell count at the implant site between the COL and LIP groups over the 7 days (considering 1, 3, and 7 days as relevant time points) with a striking increase by the latest time point in COL while LIP maintained a more physiologic trend. A detailed investigation revealed a significantly higher presence of F4‐80^+^ cells in the LIP‐treated group at 24 h (*p* < 0.001) compared to COL, suggesting an early immune activation specific to the LIP patch. Additionally, the LIP group exhibited a robust and increasing trend of CD206^+^ cells, a marker for M2‐like MΦs, over time, while the percentage of pro‐inflammatory CD86^+^ cells remained low and stable. This indicates a favorable shift toward an anti‐inflammatory, tissue‐repair phenotype. A similar trend was recently reported in an extended study by Maduka et al.^[^ ^48^ ^]^ The presence of an immunometabolic inhibitor in their investigation resulted in a decrease in the levels of pro‐inflammatory (CD86^+^CD206^−^) cells concurrently with an increase of anti‐inflammatory (or pro‐regenerative) (CD206^+^) cells in the biomaterial microenvironment. We suggest that D‐mannose may trigger a comparable immunometabolic cascade,^[^ ^49^ ^]^ as D‐mannose can compete with glucose for the same transporter and hexokinase.^[^ ^50^ ^]^ Such competitions could naturally suppress glycolysis,^[^ ^51^ ^]^ reduce mitochondrial reactive oxygen species, and so, reduce the injury‐induced pro‐inflammatory cytokine production. We propose that this may be the mechanism induced by the LIP implant surrounding the implant.

This insight highlights the unique immunomodulatory capacity of LIP, not only in the early recruitment of immune cells but also in driving a distinct innate response to the material triggered by its functionalization ^[^ ^52^ ^]^ These findings further emphasize the potential of LIP to promote a defined immune‐environment at the site of implant, aligning with the emerging understanding of MΦ dynamics as critical drivers of successful wound healing and biomaterial integration.^[^ ^48^ ^]^

At the molecular level, infiltration resulted in pronounced differences in gene expression profiles between LIP and COL, particularly concerning the healing cascade triggered by the incision site. Specifically, 20 genes were significantly downregulated in the LIP group, all of which are closely associated with pro‐inflammatory pathways, including those involved in T cell proliferation and signalling pathways mediated by NF‐κB, IL‐1α, IFN‐γ, and IL‐6. The downregulation of inflammatory pathways, coupled with the upregulation of modulatory mechanisms involved in macrophage differentiation and antimicrobial immune responses, indicates a reduced inflammatory response, consistent with the immunotuning effects observed with LIP.^[^ ^37^ ^]^ Supporting this, protein array analysis revealed elevated levels of the pro‐inflammatory cytokine IL‐1α in COL samples at 3‐ and 7‐day postimplantation, which was found minimally expressed in LIP explants. IL‐1α is known to contribute to persistent inflammasome activation impairing proper healing.^[^ ^53^ ^]^ Conversely, IL‐1 receptor antagonist (IL‐1rα) expression was significantly higher in LIP compared to COL, peaking at 7 days postimplantation. IL‐1rα, secreted by activated immune cells (i.e., MΦ, DCs, and monocytes), counteracts IL‐1‐mediated inflammation by competitively binding to the IL‐1 receptor. This anti‐inflammatory activity has been harnessed therapeutically for diabetic wound healing, with sustained administration promoting a pro‐regenerative environment.^[^ ^54^, ^55^ ^]^ Moreover, expression patterns of MIP‐1α, a key pro‐inflammatory molecule that modulates MΦ responses to implants,^[^ ^56^, ^57^ ^]^ displayed different trends between LIP and COL. Baseline expression of MIP‐1α was observed in LIP, with increased levels at day 7, indicating the ability of LIP to retain MΦ at the implant site and thereby influence the inflammatory response. Both groups exhibited a gradual reduction in monocyte chemoattractant protein 1 (MCP‐1) over time. This finding aligns with existing literature, which highlights MCP‐1 role in the early stages of wound healing by facilitating MΦ infiltration.^[^ ^58^ ^]^ The suppression of these inflammatory genes suggests that LIP reduces acute inflammation at implant site^[^ ^59^, ^60^ ^]^ and actively promotes a shift toward a more regenerative microenvironment. This trend was previously achieved by our group.^[^ ^4^ ^]^ By leveraging the immunomodulatory potential of chondroitin sulfate, we demonstrated that it is possible to accelerate the initiation of the regenerative process and activate in mammals the molecular and cellular cascades^[^ ^61^, ^62^ ^]^ activated in highly regenerative organisms (i.e., salamanders). Altogether these findings reinforce the growing evidence that controlling the inflammatory phase at early time points is crucial for optimal tissue repair.^[^ ^48^ ^]^

The wound healing array performed on explanted LIP and COL at 21 days postimplantation further validates the role of LIP in driving more favorable healing outcomes. Gene ontology analysis showed that up‐regulated genes in LIP (*Cd40lg, Col4a1, Cxcl11, Cxcl5, Fgf2, Itga1, Timp1*) are associated with ECM reorganization and tissue remodeling. Through precise biochemical and structural modifications, this patch offers a novel approach to manipulate immune cell behavior, addressing current challenges in wound healing management and providing a platform for improved therapeutic interventions. Mono‐ and polysaccharides are naturally occurring immune modulators.^[^ ^63^ ^]^ They can affect glycolytic pathways in immune cells, shifting them from a pro‐inflammatory state (often fueled by high glycolytic activity) to an anti‐inflammatory (or pro‐regenerative) state.^[^ ^64^, ^65^ ^]^ Such metabolic reprogramming is sufficient to reduce inflammation, promote tissue remodeling and maintain homeostasis.

## 5 Conclusions

Sugars, particularly monosaccharides and polysaccharides, represent a promising avenue for material functionalization due to their inherent immunotuning properties. These molecular structures have the potential to modulate immune cell metabolism, facilitating the transition from a pro‐inflammatory state to an anti‐inflammatory, tissue‐repairing phenotype. Further exploration of these moieties (including mannose) could enhance the therapeutic potential of biomaterials in tissue regeneration and immune modulation. Although much remains to be explored, this proof‐of‐concept demonstrates the considerable potential of sugar‐functionalized materials in modulating immune responses and improving therapeutic outcomes, particularly in regenerative medicine and antimicrobial applications. While integrated into collagen membranes, the mannose functionalization approach presented in this study demonstrates considerable potential as a versatile strategy that could be applied to other materials or combined with complementary therapeutics. Mannosylation not only enhances macrophage recruitment and polarization but also serves as a platform technology that can be adapted to various biomaterials, including hydrogels, polymeric scaffolds, and lipid‐based systems. By harnessing mannose's interaction with the MRC‐1 on MΦ, this approach enables the creation of localized immune‐modulating environments across a range of applications, from wound healing to implantable devices.

## Conflict of Interest

The authors declare no conflict of interest.

## Author Contributions

F.T. and B.C., conceived the idea and planned the experiments. F.T., with the help of J.O.M. and L.P. synthesized and characterized the materials and run the in vivo experiments. B.C., with the support of S.M. and X.W. perform the in vitro experiments and the molecular evaluation of the in vivo study. C.C. and J.E.R. performed the proteomic analysis. S.K. and C.A. performed the bacteria experiments. E.T., F.T. and B.C. supervised the study and provided funding. B.C. took the lead in writing the manuscript with the help of F.T. and All authors provided critical feedback and helped shape the research, analysis and manuscript.

## Supporting information



Supporting Information

## Data Availability

The data that support the findings of this study are available from the corresponding author upon reasonable request.
