# Supplementary material for: Sugar Functionalized Collagen Material for Local Modulation of Innate Immunity
Source: Adv Sci (Weinh). 2025 May 24;12(31):2415364. doi: 10.1002/advs.202415364 (PMC12376565; doi:10.1002/advs.202415364)
Supplement: Supplementary file 1 — Supporting Information [file ADVS-12-2415364-s001.docx]

**SUPPLEMENTARY INFORMATION**

**SUPPLEMENTARY FIGURES**

**
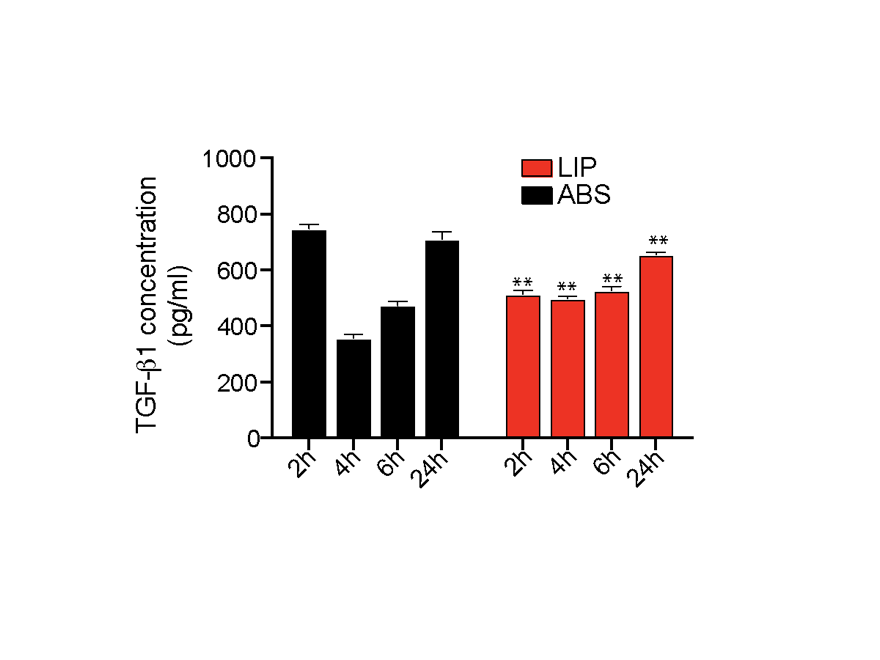
**

**Supplementary Figure S1.** Quantification of TGF-β levels released over time by MΦ grown onto ABS and LIP using ELISA Data are presented as mean ± SD from 3 independent experiments. Statistical significance: p<0.01.


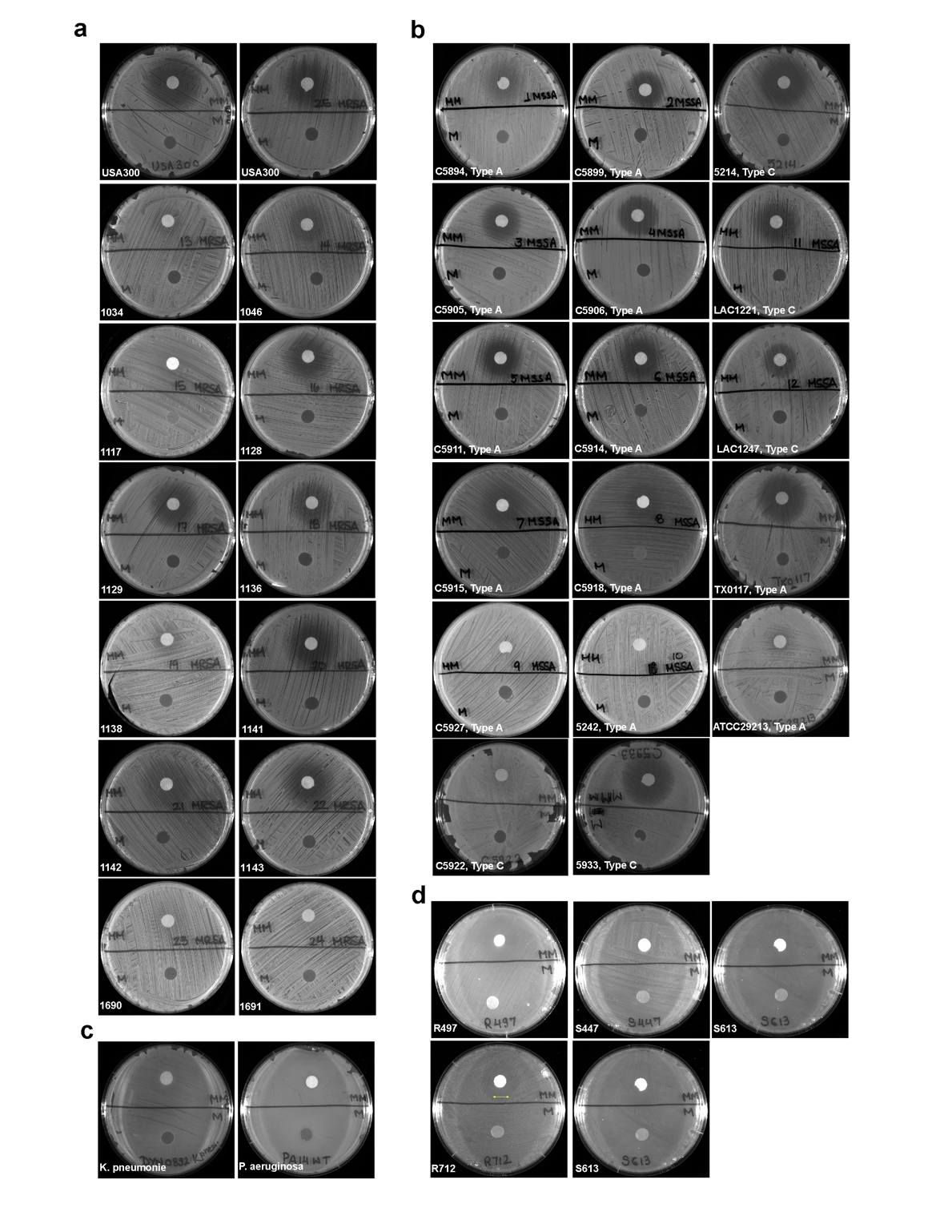


**Supplementary Figure S2.** Growth inhibitory effect of LIP on bacterial cultures. This figure illustrates the inhibitory effects of LIP (top) and COL (bottom) on bacterial growth. Disks were applied to bacterial cultures and antimicrobial subinhibitory zones against MRSA (**a**, n=13), MSSA (**b**, n=17), *K. pneumonia/P. aeruginosa*(**c**), and *E. faecium/faecalis* (**d**, n=3 and n=2, respectively) strains were measured after a 24-hr incubation period at 37°C. The size (diameter in mm) of the inhibition zones, indicated by clear areas surrounding the disks, reflects the effectiveness of each material in hindering bacterial growth. MRSA strain USA 300 was repeated twice, as shown in the figure.


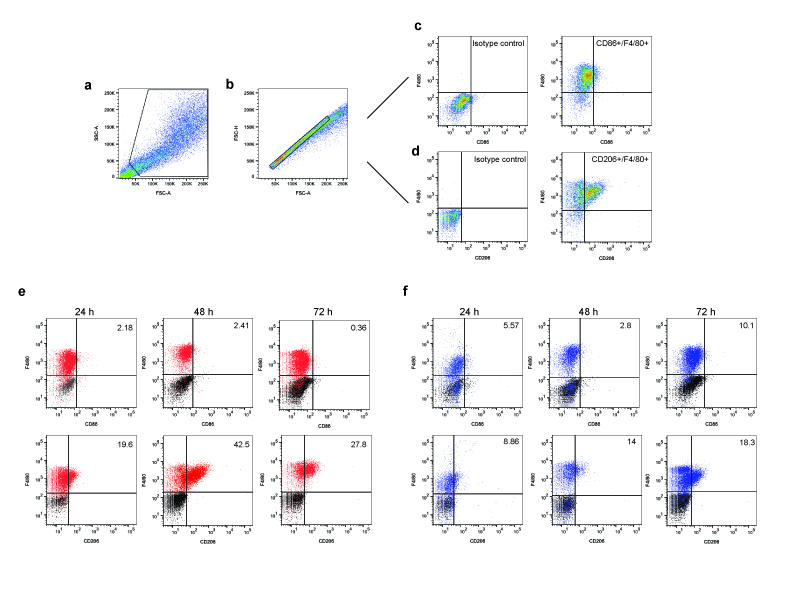


**Supplementary Figure S3.** Flow cytometry gating strategy for identifying macrophage populations and quantification overtime. (**a**) Morphological plot displaying forward scatter (FSC) and side scatter (SSC) parameters used to define the cell population of interest, (**b**) Singlet gating to exclude doublets and ensure analysis of individual cells. (**c**) Quadrant plot displaying F4/80^+^ macrophages and their expression of CD86, indicative of pro inflammatory M1-like macrophages. (**d**) Quadrant plot illustrating F4/80^+^ macrophages and their expression of CD206, indicative of M2-like macrophages. Cell populations identified using isotype controls are also shown as a reference for setting gating thresholds. Plots showing analysis of cell populations infiltrating LIP (**e**) and COL (**f**) overtime (24, 48, and 72 h).

**SUPPLEMENTARY TABLE**

**Supplementary Table S1.** Proteome Profiler Array kit at designated time points (24 h, 72 h, and 7 days). Protein expression levels are shown for key cytokines, chemokines, and other inflammatory mediators. The two groups include COL and LIP explants. Expression values are represented as relative intensities normalized to control samples, with higher values indicating greater protein expression.

| **Cytokine** | **COL** | | | **LIP** | | |
| --- | --- | --- | --- | --- | --- | --- |
|  | **24 h** | **72 h** | **7 days** | **24 h** | **72 h** | **7 days** |
| BLC/CXCL13/BCA-1 | 0.0095 | 0.072 | 0.0245 | 0.0285 | 0.0485 | 0.0115 |
| C5/C5a | 0.0205 | 0.272 | 0.193 | 0.0055 | 0.072 | 0.0115 |
| G-CSF | 0.0125 | 0.0095 | 0.0015 | -0.001 | 0.002 | 0.002 |
| GM-CSF | 0 | 0.002 | -0.0025 | 0 | 0.001 | -0.0015 |
| I-309 | 0.001 | 0.006 | 0.0005 | -0.0005 | 0.0045 | 0.0015 |
| Eotaxin/CCL21 | 0.0005 | -0.001 | -0.0015 | -0.0005 | 0.0015 | -0.002 |
| ICAM-1 | -0.001 | -0.003 | -0.003 | -0.0005 | 0.001 | -0.002 |
| IFN-γ | 0.0005 | 0.0005 | 0.001 | 0.001 | 0.0055 | 0.0005 |
| IL-1α | 0.001 | 0.2415 | 0.014 | 0.0005 | 0.008 | 0.0195 |
| IL-1β | 0.031 | 0.575 | 0.508 | 0.0075 | 0.0055 | 0.2355 |
| IL-1rα | 0.077 | 0.986 | 0.383 | 0.1405 | 0.3535 | 0.768 |
| IL-2 | -0.0005 | 0.01 | 0.016 | -0.0005 | 0.023 | 0.035 |
| IL-3 | -0.0005 | 0.009 | -0.0005 | -0.001 | 0.005 | 0.001 |
| IL-4 | -0.0005 | 0.009 | 0.0055 | -0.002 | 0.006 | -0.002 |
| IL-5 | -0.0005 | 0.0015 | -0.0005 | -0.0005 | 0.0005 | -0.002 |
| IL-6 | 0.0005 | 0.061 | -0.0005 | -0.0025 | 0.0005 | -0.0025 |
| IL-7 | 0.0005 | 0.0115 | 0.0015 | -0.0005 | 0.004 | 0.001 |
| IL-10 | -0.0005 | 0.002 | 0.0005 | 0 | 0.003 | 0.001 |
| IL-13 | -0.001 | 0.006 | 0.0055 | 0 | 0.009 | 0.0055 |
| IL-12p70 | 0 | 0.0035 | -0.002 | -0.001 | 0 | -0.0025 |
| IL-16 | 0.009 | 0.1555 | 0.064 | 0.005 | 0.0245 | 0.0675 |
| IL-17 | 0.0015 | 0.028 | 0.036 | -0.002 | 0.0015 | 0.04 |
| IL-23 | 0.046 | 0.0585 | 0.1905 | 0.0015 | 0.025 | 0.2625 |
| IL-27 | -0.001 | 0.007 | 0.0075 | 0.001 | 0.008 | 0.0115 |
| IP-10 | 0.477 | 1.1 | 0.874 | 0.134 | 0.9105 | 0.341 |
| I-TAC | 0.013 | 0.035 | 0.0215 | 0 | 0.0235 | 0.0015 |
| KC | 0.0125 | 0.684 | 0.02 | 0.0105 | 0.0235 | 0.023 |
| M-CSF | 0.0135 | 0.039 | 0.017 | 0.0085 | 0.0175 | 0.0195 |
| JE | 0.5225 | 1.0425 | 0.5425 | 0.3155 | 0.869 | 0.6025 |
| MCP-5 | 0.031 | 0.0765 | 0.014 | 0.023 | 0.0285 | 0.0135 |
| MIG | 0.0055 | 0.0255 | 0.003 | 0.002 | 0.013 | 0.0105 |
| MIP-1α | 0.114 | 0.914 | 0.432 | 0.0185 | 0.177 | 0.3535 |
| MIP-1β | 0.012 | 0.7185 | 0.0125 | 0 | 0.0075 | 0.0085 |
| MIP-2 | 0.1495 | 0.8995 | 0.1395 | 0.014 | 0.0025 | 0.1075 |
| RANTES | 0.0265 | 0.4725 | 0.115 | -0.001 | 0.0215 | 0.021 |
| SDF-1 | 0.0115 | 0.2065 | 0.1875 | 0.0115 | 0.093 | 0.428 |
| TARC | -0.0025 | 0.0015 | -0.0015 | 0 | 0 | -0.0015 |
| TIMP-1 | 0.064 | 0.3835 | 0.1915 | 0.026 | 0.3085 | 0.237 |
| TNF-α | 0.007 | 0.232 | 0.0135 | 0.0025 | 0.01 | 0.018 |
| TREM-1 | 0.065 | 0.3055 | 0.146 | 0.0375 | 0.093 | 0.092 |
